# GPi‐DBS for *SERAC1* ‐Related Dystonia‐Parkinsonism

**DOI:** 10.1002/mdc3.70332

**Published:** 2025-08-30

**Authors:** Vidal Yahya, Alessandro Di Maio, Mauro Treddenti, Ludovica Tundo, Giulia Scacciatella, Costanza Masetti, Marta Pengo, Chiara Casellato, Desiree Lattanzi, Marcello Egidi, Sara Marceglia, Tommaso Bocci, Alberto Priori, Gaia Oggioni

**Affiliations:** ^1^ Aldo Ravelli Center for Neurotechnology and Experimental Brain Therapeutics, Department of Health Sciences University of Milan Milan Italy; ^2^ Neurology Unit, ASST Santi Paolo e Carlo San Paolo University Hospital Milan Milan Italy; ^3^ Neurosurgery Unit, ASST Santi Paolo e Carlo, San Carlo Borromeo Hospital Milan Italy

**Keywords:** *SERAC1*, dystonia, GPi, DBS

Globus pallidus internus deep brain stimulation (GPi‐DBS) is an effective treatment for dystonia. Yet, the outcome of GPi‐DBS is variable in monogenic dystonia: optimal responses are commonly reported for DYT‐*TOR1A* and ‐*SGCE*, a significant benefit is also recognized for DYT‐*GNAL*, ‐*KMT2B*, ‐*PANK2*, ‐*TAF1*, and ‐*THAP1*, while the effect of GPi‐DBS remains uncertain for rarer forms.^1^, ^2^, ^3^

In this context, dystonia associated with monogenic mitochondriopathies constitutes a challenging field characterized by significant genetic heterogeneity and complex clinical pictures.^4^ GPi‐DBS implantation is approached very carefully in this cohort for several reasons: basal ganglia, including GPi, are particularly susceptible to mitochondrial dysfunction^5^; systemic disturbances classically associated with mitochondrial diseases may interfere with neuroimaging and surgery (eg, cochlear implants for sensorineural deafness); intellectual disability or young age often contraindicate awake surgery; common anesthetics like propofol suppress mitochondrial activity, potentially causing harm.^6^

A rare form of dystonia lacking solid evidence on GPi‐DBS is caused by biallelic *SERAC1* variants. *SERAC1*‐related disease is a mitochondriopathy encompassing a wide phenotypic spectrum, ranging from severe infantile‐onset Leigh‐like syndromes (ie, 3‐methylglutaconic aciduria with deafness, encephalopathy, and Leigh‐like syndrome—*MEGDEL*) to the recently described adult‐onset dystonia‐parkinsonism.^7^, ^8^, ^9^ Typically, *SERAC1*‐related dystonia involves cranio‐cervical districts, manifesting with dysarthria, dysphagia, repetitive tongue protrusion, and excessive drooling.^7^

Here we report a patient with *SERAC1*‐related dystonia treated with GPi‐DBS. The proband, whose genetic and clinical features are detailed in reference,^9^ is a 56‐year‐old female carrying the homozygous c.1231C>T p.(Arg411Cys) variant. She presented dystonia‐parkinsonism with onset at 38 years, representing one of the mildest *SERAC1*‐related phenotypes reported to date.^9^ After the diagnosis, the patient underwent several therapeutic attempts: levodopa (up to 1000 mg/day), amantadine, trihexyphenidyl, and tetrabenazine were administered with poor response; onabotulinum toxin A (right splenius capitis 75 U, right levator scapulae 30 U, left platysma 20 U, upper orbicularis oris 2.5 U, intrinsic muscles of the tongue 10 U), clozapine (suspended due to neutropenia), and olanzapine offered a relevant but partial clinical benefit (Video 1).

**VIDEO 1:** Neurological examination before DBS implantation.

Thus, at the age of 55 years, the patient underwent DBS surgery. The surgical procedure was performed under general anesthesia using the Leksell Vantage stereotactic system and the Medtronic StealthStation S8, guided by 1.5 T brain MRI and CT imaging (Fig. 1). The anterior commissure‐posterior commissure (AC‐PC) measured 23.2 mm. The optic tract was traced and its lateral coordinate—identified on a coronal plane 2 mm anterior to the midcommissural point—was selected as the lateral coordinate for the trajectory targeting the GPi. Based on microelectrode recordings of GPi activity from 3 to 1 mm above the target across all trajectories and absence of pyramidal effects after macrostimulation up to 3 mA, two Medtronic® B3300542 directional leads were implanted bilaterally. The lead tips were positioned 1 mm above the target along the central track and connected to the Medtronic Percept PC neurostimulator. Based on the planned coordinates and final electrode placement, the stimulation site was estimated to lie 2–3 mm anterior and 22 mm lateral at the AC‐PC plane (Video 2). Two months later, stimulation was progressively initiated reaching the following setting at 6 months follow‐up: right GPi 10‐C+ 3.2 mA, 90 μs, 110 Hz; left GPi 2‐C+ 3.2 mA, 90 μs, 110 Hz.

**FIG 1:**
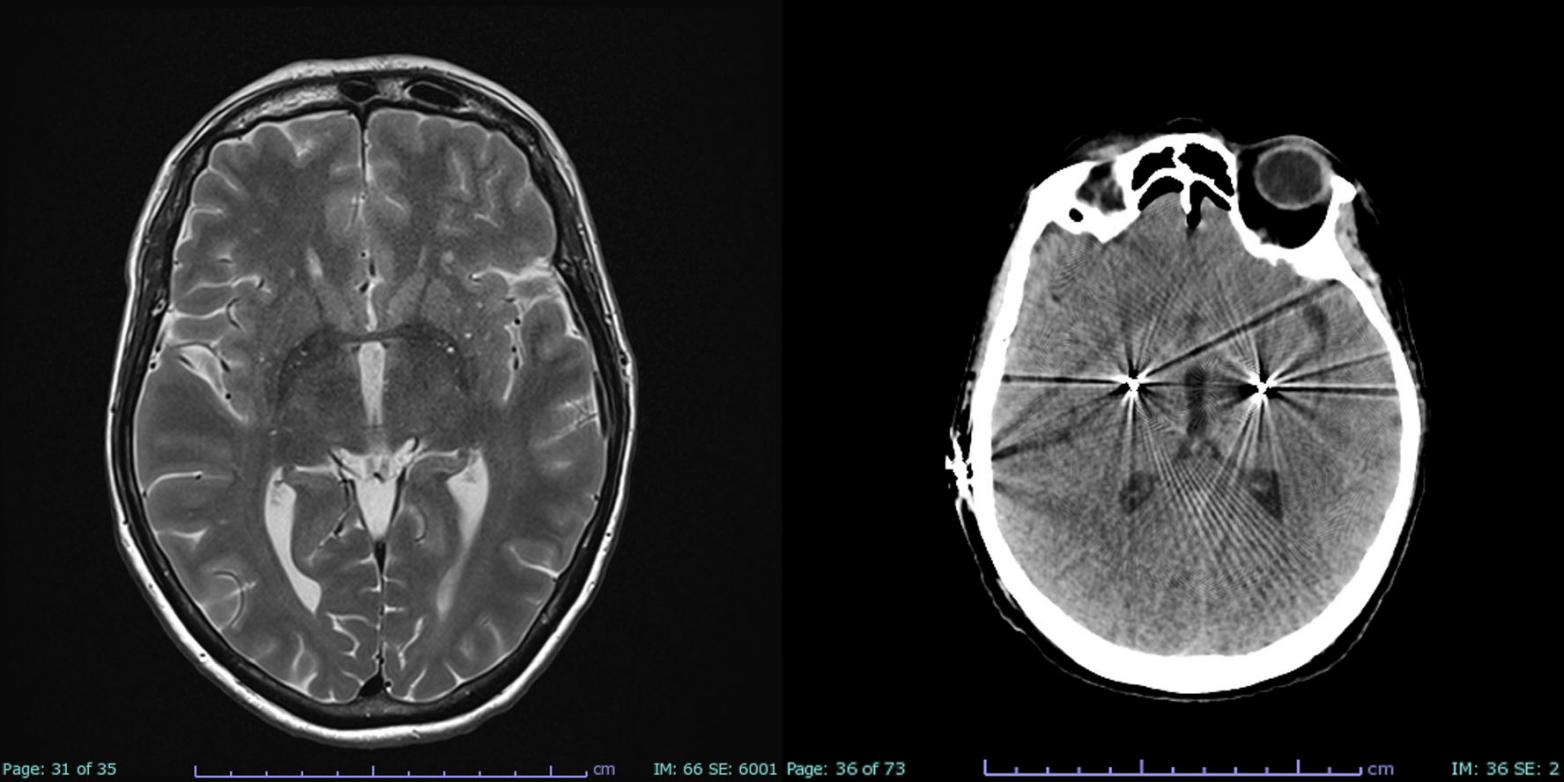
Brain MRI and CT after bilateral GPi‐DBS implantation.

**VIDEO 2:** Neurological examination after DBS implantation, off stimulation.

The patient exhibited a remarkable improvement with GPi‐DBS: speech, though still dysarthric, became eventually comprehensible; jaw tremor and tongue protrusion improved significantly; cervical posture appeared stable, yet with resolution of the chronic pain; bradykinesia as well as rest and action tremor almost disappeared in right upper limb and became milder in left upper limb; repetitive actions execution became more fluent (Video 3). No dysphagia could be detected at instrumental evaluation. No neuropsychological abnormalities were detected besides a mild attentional deficit.

**VIDEO 3:** Neurological examination after DBS implantation, on stimulation.

Clinical response to GPi‐DBS has been quantitatively evaluated with the Movement Disorders Society Unified Dystonia Rating Scale (40 → 19), the Movement Disorders Society Global Dystonia Severity Scale (35 → 13), the Burke‐Fahn‐Marsden Movement Scale (41 → 21) – Supplementary File S1. Health‐related Quality of Life Instrument with 20 items (HINT‐20) total transformed score improved from 60/100 to 85/100.

The resolution of dysphagia allowed the resumption of an adequate oral intake leading to weight gain from 56 to 75 kg (BMI 20.1 → 26.9 kg/m^2^) in 6 months, therefore feeding through percutaneous endoscopic gastrostomy was suspended. As mild cervical dystonia persisted, onabotulinumtoxin A was injected again in the right splenius capitis (75 U) and in the left trapezius (25 U). The global motor and psychological improvement allowed a gradual tapering of pharmacological therapy with olanzapine.

To our knowledge, among the ~70 reported cases of *SERAC1*‐related disease, only a 16‐year‐old girl with generalized dystonia, athetoid movements, and ataxia underwent GPi‐DBS with significant and enduring motor improvement.^10^

In conclusion, we report a patient with *SERAC1*‐related dystonia‐parkinsonism who had a remarkable and persistent benefit from bilateral GPi‐DBS. Our case, along with the one reported by Sharma et al, 2021^10^ suggests that GPi‐DBS can be a valuable therapeutic option for this rare disease, especially for patients with later onset and mild phenotype, emphasizing the importance of *SERAC1* screening in patients with dystonia.

## Author Roles

(1) Research project: A. Conception, B. Organization, C. Execution; (2) Statistical Analysis: A. Design, B. Execution, C. Review and Critique; (3) Manuscript Preparation: A. Writing of the first draft, B. Review and Critique

V.Y.: 1A, 1C, 3A.

A.D.M.: 1A, 1C, 3A.

M.T.: 1C, 3B.

L.T.: 1C, 3B.

G.S.: 1C, 3B.

C.M.: 1C, 3B.

M.P.: 1C, 3B.

C.C.: 1C, 3B.

D.L.: 1C, 3B.

M.E.: 1B, 1C, 3A.

S.M.: 1C, 3B.

T.B.: 1C, 3B.

A.P.: 1A, 1B, 3B.

G.O.: 1A, 1C, 3A.

## Disclosures

**Ethical Compliance Statement:** The authors confirm that the approval of an institutional review board was not required for this work. Written informed consent was obtained by the patient. We confirm that we have read the Journal's position on issues involved in ethical publication and affirm that this work is consistent with those guidelines.

**Funding Sources and Conflicts of Interest:** No specific funding was received for this work. The authors declare that there are no conflicts of interest relevant to this work.

**Financial Disclosures for the previous 12 months:** AP was consultant for Newronika, Milan, Italy SpA in the last 12 months. The other authors declare that there are no additional disclosures to report.

## Supporting information


**File S1.** Tables displaying detailed motor scales before and after DBS surgery.

## Data Availability

The data that support the findings of this study are available on request from the corresponding author. The data are not publicly available due to privacy or ethical restrictions.
