# Supplementary material for: GPi‐DBS for SERAC1 ‐Related Dystonia‐Parkinsonism
Source: Mov Disord Clin Pract. 2025 Aug 30;13(2):568–71. doi: 10.1002/mdc3.70332 (PMC13020567; doi:10.1002/mdc3.70332)
Supplement: Supplementary file 4 — File S1. Tables displaying detailed motor scales before and after DBS surgery. [file MDC3-13-568-s001.docx]

**Motor scales before and after DBS surgery (stimulation ON)**

Table 1: UDRS before and after DBS surgery (stimulation ON). *https://www.movementdisorders.org/MDS/MDS-Rating-Scales/Unified-Dystonia-Rating-Scale-UDRS-.htm*

| Region | **Before DBS surgery** | | | **After DBS surgery (stimulation ON)** | | |
| --- | --- | --- | --- | --- | --- | --- |
|  | Duration | Motor Severity | **Product** | Duration | Motor Severity | **Product** |
| Eyes and upper face | - | - | **0** | - | - | **0** |
| Lower face | 3.5 | 2 | **7** | 3.5 | 1 | **3.5** |
| Jaw and tongue | 3.5 | 2 | **7** | 3.5 | 1 | **3.5** |
| Larynx | - | - | **0** | - | - | **0** |
| Neck | 3.5 | 2 | **7** | 3.5 | 1 | **3.5** |
| Shoulder and proximal arm (R and L) | 3.5 | 2 | **7** | 3.5 | 1 | **3.5** |
| Distal arm and hand including elbow  (R and L) | 3.5 | 3 | **10.5** | 3.5 | 1 | **3.5** |
| Pelvis and proximal leg (R and L) | - | - | **0** | - | - | **0** |
| Distal leg and foot including knee  (R and L) | 1.5 | 1 | **1.5** | 1.5 | 1 | **1.5** |
| Trunk | - | - | **0** | - | - | **0** |
| Total | **40** | | | **19** | | |

Table 2: GDS before and after DBS surgery (stimulation ON). *https://www.movementdisorders.org/MDS/MDS-Rating-Scales/Global-Dystonia-Scale-GDS.htm*

| Region | **Before DBS surgery** | **After DBS surgery (stimulation ON)** |
| --- | --- | --- |
| Eyes and upper face | 0 | 0 |
| Lower face | 5 | 2 |
| Jaw and tongue | 8 | 2 |
| Larynx | 0 | 0 |
| Neck | 6 | 2 |
| R Shoulder and proximal arm | 2 | 1 |
| L Shoulder and proximal arm | 0 | 0 |
| R Distal arm and hand including elbow | 6 | 2 |
| L Distal arm and hand including elbow | 6 | 2 |
| R Pelvis and proximal leg | 0 | 0 |
| L Pelvis and proximal leg | 0 | 0 |
| R Distal leg and foot including knee | 0 | 0 |
| L Distal leg and foot including knee | 2 | 2 |
| Trunk | 0 | 0 |
| Total | **35** | **13** |

Table 3: BFMMS before and after DBS surgery (stimulation ON).
*Burke RE, Fahn S, Marsden CD, Bressman SB, Moskowitz C, Friedman J. Validity and reliability of a rating scale for the primary torsion dystonias. Neurology. 1985 Jan;35(1):73-7. doi: 10.1212/wnl.35.1.73. PMID: 3966004.*

| Region | **Before DBS surgery** | | | | **After DBS surgery (stimulation ON)** | | | |
| --- | --- | --- | --- | --- | --- | --- | --- | --- |
|  | Provoking Factor | Severity Factor | Weight | **Product** | Provoking Factor | Severity Factor | Weight | **Product** |
| Eyes | - | - | - | **0** | - | - | - | **0** |
| Mouth | 4 | 3 | 0.5 | **6** | 4 | 2 | 0.5 | **4** |
| Speech and swallowing | 4 | 4 | 1 | **16** | 2 | 2 | 1 | **4** |
| Neck | 4 | 3 | 0.5 | **6** | 4 | 2 | 0.5 | **4** |
| R Arm | 2 | 3 | 1 | **6** | 2 | 2 | 1 | **4** |
| L Arm | 2 | 3 | 1 | **6** | 2 | 2 | 1 | **4** |
| Trunk | - | - | - | **0** | - | - | - | **0** |
| R Leg | - | - | - | **0** | - | - | - | **0** |
| L Leg | 1 | 1 | 1 | **1** | 1 | 1 | 1 | **1** |
| Total | **41** | | | | **21** | | | |
